# Evolutionary ecomorphology for the twenty-first century: examples from mammalian carnivores

**DOI:** 10.1098/rspb.2023.1400

**Published:** 2023-11-29

**Authors:** Julia A. Schwab, Borja Figueirido, Alberto Martín-Serra, Julien van der Hoek, Therese Flink, Anne Kort, Juan Miguel Esteban Núñez, Katrina E. Jones

**Affiliations:** ^1^ Department of Earth and Environmental Sciences, University of Manchester, M13 9PL Manchester, UK; ^2^ Departamento de Ecología y Geología, Facultad de Ciencias, Universidad de Málaga, Málaga, Spain; ^3^ Department of Palaeobiology, Swedish Museum of Natural History, PO Box 50007, 10405 Stockholm, Sweden; ^4^ Department of Earth and Atmospheric Sciences, Indiana University Bloomington, 1001 E 10th St, Bloomington, IN, USA; ^5^ Department of Earth and Environmental Sciences, University of Michigan, 1100 N University Ave, Ann Arbor, MI 48109, USA

**Keywords:** carnivora, ecomorphology, geometric morphometrics

## Abstract

Carnivores (cats, dogs and kin) are a diverse group of mammals that inhabit a remarkable range of ecological niches. While the relationship between ecology and morphology has long been of interest in carnivorans, the application of quantitative techniques has resulted in a recent explosion of work in the field. Therefore, they provide a case study of how quantitative techniques, such as geometric morphometrics (GMM), have impacted our ability to tease apart complex ecological signals from skeletal anatomy, and the implications for our understanding of the relationships between form, function and ecological specialization. This review provides a synthesis of current research on carnivoran ecomorphology, with the goal of illustrating the complex interaction between ecology and morphology in the skeleton. We explore the ecomorphological diversity across major carnivoran lineages and anatomical systems. We examine cranial elements (skull, sensory systems) and postcranial elements (limbs, vertebral column) to reveal mosaic patterns of adaptation related to feeding and hunting strategies, locomotion and habitat preference. We highlight the crucial role that new approaches have played in advancing our understanding of carnivoran ecomorphology, while addressing challenges that remain in the field, such as ecological classifications, form–function relationships and multi-element analysis, offering new avenues for future research.

## 1. Introduction

Ecomorphology is broadly defined as the relationship between an organism's physical form (morphology) and its ecological roles within its environment [1]. It has been a long-standing interest of evolutionary biologists to explain variation in morphological structure in relation to ecology, habitat and environment (e.g. [2,3]). However, the field has itself undergone radical evolution. Beginning as a qualitative science, visually comparing species and patterns, it has become increasingly quantitative (e.g. [4,5]). For example, the application of traditional morphometrics such as linear or angular measurements allowed quantification of patterns observed by eye. Further, in the last 15 years, new quantitative methods such as geometric morphometrics (GMM) and multivariate statistics have spurred a revolution in the field, with more comprehensive and subtle shape variations distinguishable and the ability to quantify such variations in association with ecotypes. GMM differs from traditional morphometrics as it uses point coordinates (landmarks) to define shape and can therefore quantify more complex and subtle morphological variation. Thus, the ‘GMM revolution’ of the early 2000s provided a way, for the first time, to quantify complex variations in biological shape in two and three dimensions [6].

Ecomorphology is a key area of research in evolutionary biology, ecology, palaeontology and comparative anatomy; and can be used to reconstruct and understand ecological variation today and through deep time. One goal is to understand the lifestyles of extinct taxa by comparing their skeletal morphology with their modern relatives, which can provide insights into the evolution of species and how they have adapted to their environments over time (e.g. [7,8]). Ecomorphological studies can also clarify the relationships between form, function, and environment, shed light on the mechanisms of evolution and adaptation, and inform conservation efforts (e.g. [9]). By examining the relationships between morphology and ecology, ecomorphologists can reveal the adaptations that enable species to survive and thrive in their environments. This information can then be used to help identify conservation priorities and inform the design of habitats and other interventions aimed at preserving biodiversity. A related concept, morphodynamics, disentangles the relationship between evolutionary factors such as phylogeny, evo-devo, environment and function in morphological evolution [10]. Though very promising, difficulties around defining complex ecological categories can often make comparing and interpreting ecomorphological studies problematic and remains an ongoing challenge in the field [11] (see more discussion in 3). In summary, ecomorphology plays a critical role in our understanding of the evolution and ecology of organisms and the ways they interact with their environment [1].

**Figure 1.**
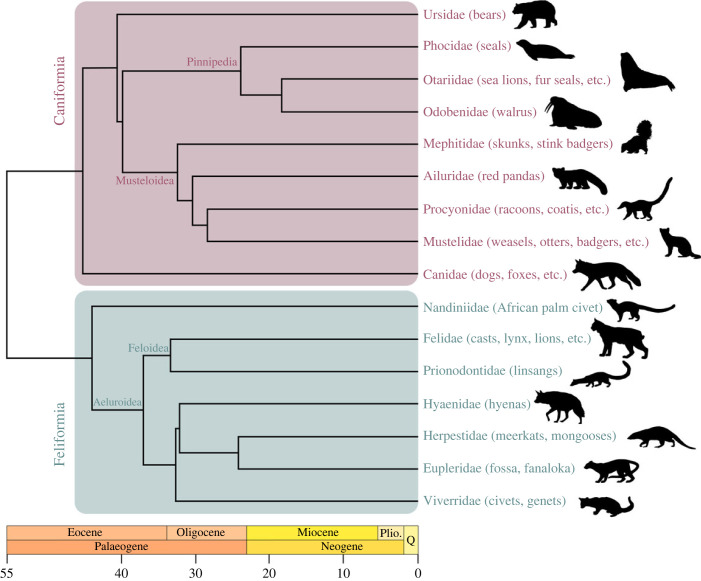
Phylogeny of major carnivoran groups, based on the work of Nyakatura & Bininda-Emonds [12]. Silhouettes not to scale. Sourced from Phylopic.org, all under public domain.

Carnivora is an order of placental mammals that includes many carnivorous species (e.g. cats, dogs, hyenas; figure 1), but also a wide diversity of other ecologies. They are an ideal model system for ecomorphology research due to their diverse ecological roles, adaptations to different environments and evolutionary relationships. They exhibit a wide range of morphological and behavioural adaptations that have allowed them to occupy a range of ecological niches, from terrestrial to arboreal to aquatic, and from small to large body sizes. This variety of adaptations provides an excellent opportunity to study the relationships between form and function, and to better understand the evolutionary processes. Additionally, carnivorans are a well-studied group with a well-resolved phylogeny [12], making it easier to link evolutionary relationships with morphological adaptations (figure 1). These features make carnivorans an ideal group for exploring ecomorphological questions and advancing our understanding of the interplay between ecology and anatomy, and how this relationship evolved through deep time. Additionally, the fossil record of carnivorans is well-documented and extends back to the Late Cretaceous period (about 65 Ma) [13]. This allows researchers to study the evolution of carnivoran morphologies and their adaptations over a long period of time. These factors make it possible to test a range of hypotheses about the evolution of form and function in these animals, leading to a better understanding of adaptive evolution.

The goal of this review is to demonstrate the utility and rich history of quantitative ecomorphological studies in carnivorans and provide a case study for the trajectory of the field more broadly. We focus on the recent explosion of GMM work in carnivoran ecomorphology and its impact on (a) feeding and foraging (skull and jaw), (b) observing the environment (inner ear labyrinth, nose and brain endocast), and (c) locomotion and hunting (limbs and axial skeleton). Finally, we summarize the major outstanding questions in carnivore ecology, and argue for a future approach across anatomical systems, which can provide a more holistic understanding of ecomorphological adaptation.

## 2. Overview of carnivoran ecomorphology

### (a) Feeding and foraging

**Figure 2.**
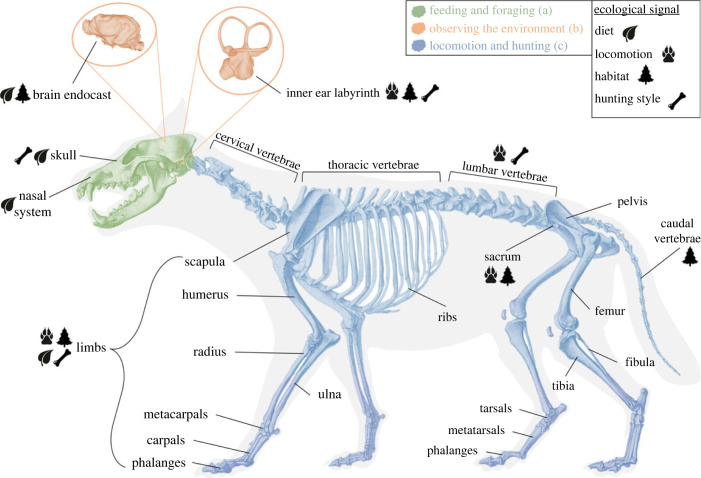
Carnivoran skeleton highlighting different parts associated with sections in the text. Section 2a: feeding and foraging (in green); section 2b: observing the environment (in orange); section 2c: locomotion and hunting (in blue). Symbols indicate ecological signal for different skeletal systems. Inner ear labyrinth shown resembles *Canis lupus* and the brain endocast *Crocuta crocuta*.

Obtaining and processing food is vital to an organism's survival and exerts a strong selective pressure on populations. Further, the evolution of feeding systems is intimately linked to coevolution of animals with their environment, as the availability and readiness of food sources may vary through time or in response to climatic change [14]. Therefore, there has been strong interest in revealing ecomorphological indicators of feeding behaviour and foraging in the carnivoran skull and jaw. Feeding behaviours in carnivores vary widely from carnivorous to herbivorous, omnivorous and insectivorous forms [15] (figure 2). While much of this early work has focused on linear measures, the skull and jaw were among the earliest anatomical system to be studied using GMM.

#### (i) Jaw

The bones of the jaw exhibit a great diversity of shape and size that reflect the functional demands placed on them during chewing, biting and hunting (e.g. [16,17]). The shape and size of the carnivoran mandible are closely related to ecological adaptations, with generalists displaying more convergent mandibular shapes than specialists [18]. For example, some species of carnivorans have large, heavy jaw bones that provide a strong base for the jaw muscles, allowing them to generate powerful bites, whereas other species have lighter, more slender jaw bones, allowing for greater speed and agility in pursuit of prey [19–22]. This reflects a trade-off between gape and bite force. Hypercarnivorous species show mandibular adaptations to bite force and gape angle. Sabretoothed cats are an exception, as their hypertrophied upper canines resulted in an increase in jaw gape which reduced bite force [23], leading to an increased risk of extinction [24]. However, sabretooth taxa also show similarities to non-sabretooth forms [25]. This is directly related to prey capture, as solitary ambush predators usually kill with a single bite compared to pack hunters (figure 1; canids, hyaenids) [20].

#### (ii) Skull

Skull shape varies in response to functional demands that can be correlated with diet [26–29]. Carnivorans exhibit a wide range of ecomorphological adaptations in their skulls, reflecting their diverse ecological niches, hunting styles and feeding ecologies. Hunting behaviour in carnivorans also depends on the predator body mass [30]. For example, smaller species (less than 15–20 kg) are usually specialists of very small vertebrates and invertebrates, which weigh much less than the predator body mass [30,31]. By contrast, larger species (greater than 15–20 kg) are large-vertebrate prey specialists with similar or smaller body mass than prey species. Therefore, those species surpassing the approximately 20 kg of physiological threshold [32] just predate upon prey larger than themselves, which is also reflected in their craniodental skeletons [33,34]. For example, large-prey specialist felids possess stouter canines and incisors and wider snouts than small and mixed prey feeders because these traits are useful for delivering the stranglehold killing-bite on large prey that cats employ [35].

In Felidae (mostly solitary hunters; figure 1), a larger skull size corresponds to an increased gape, but a decrease in bite force [21]. While in canids (often pack hunters), upper jaw dimensions vary based on diets, with an inverse relationship between upper jaw length and bite force [20]. Similarly, herbivorous carnivorans exhibit higher bite forces, to be able to chew on tough foods, which parallels the evolution of the hypercarnivorous bone-cracking species such as hyaenids [36] or highly derived borophagine dogs [37–39]. Mustelids have more robust skulls (shortening of the rostrum and broadening of the mastoid zygomatic arch breadth) through the Mid-Miocene climatic shift [40]. This can be associated with increase in relative bite force [41,42] and larger jaw muscles counteract smaller body sizes. Mustelids and felids have the most powerful bites and canids the weakest among the four family groups [41,43,44]. The craniodental morphology in ursids shows adaptations to their feeding ecologies, herbivore versus carnivore with omnivore being intermediate [4,5].

#### (iii) Pinnipeds

Secondarily aquatic carnivorans, the pinnipeds (seals and kin, figure 1) have also adapted to the challenge of feeding in the aquatic environment in a variety of ways [45]. They are adept at both biting and suction, using one or both behaviours depending on their requirements [45], and have craniodental adaptations related to predatory behaviour and targeted prey (e.g. [45–48]). Relative to terrestrial species, pinniped skull disparity increased by exploring new skull shapes without increases in evolutionary rate that would indicate an adaptive radiation [46]. On the other hand, pinnipeds underwent a change in mandibular function from carnivore-like chewing to other types of feeding such as filtering or grip-and-tear behaviour [47]. Pinnipeds are, therefore, a remarkable example of adaptation, as they have successfully overcome the challenges posed by the marine realm.

### (b) Observing the environment

Sensory systems allow organisms to successfully navigate their environment, hunt prey, escape danger and interact with other members of their species. While vertebrate sensory systems are numerous and well developed, the major focus of research has been on systems which are housed within the cranial skeleton, and therefore have potential for interpretation in the fossil record. These include the inner ear labyrinth, which is involved in the senses of hearing, balance and equilibrium, the nasal system, involved in smell and respiration, and the brain endocast, which can provide insights into multiple sensory systems such as vision, olfaction and movement (figure 2).

#### (i) Inner ear labyrinth

The inner ear labyrinth is associated with the sense of balance and equilibrium and can be used for the detection of head movements and gaze stabilization [49], which makes it an ideal system to use for reconstructing the lifestyles and behaviour of animals. Located in the petrosal bone it represents one of the best-preserved anatomical systems in the fossil record. The membranous system is enclosed by the bony labyrinth to form the inner ear. The size and morphology of the soft tissue is thought to be mirrored by its bony cavity and hence can be used to compare fossils with their extant relatives to draw inferences about their ecomorphological adaptations (e.g. [8,50–52]). The inner ear labyrinth can be divided into two systems. The vestibular system consisting of three semicircular canals responsible for angular acceleration and the vestibule containing the utricle and saccule for linear acceleration, while the auditory system comprises of the coiled cochlea associated with the sense of hearing.

In carnivorans, there are several studies associating bony labyrinth morphology with both phylogenetic relationships, but also ecomorphological aspects. In mustelids (weasels, otters and kin), both the vestibular system and the cochlea are associated with ecology, providing a strong signal for semi-aquatic lifestyle, as well as for terrestrial, semi-arboreal, arboreal and semi-fossorial [53]. The bony labyrinth of Canidae and Feloidea varies based on their hunting strategies (pursuit, pounce, ambush and occasional) [8], with species using faster modes of locomotion (e.g. wolf, leopards) showing larger semicircular canals compared to slower taxa. Cheetahs are extreme high-speed runners and have developed a highly specialized inner ear morphology [54]. In aquatic carnivorans, the bony labyrinth responds to their aquatic behaviour, with a reduction in the diameters of the semicircular canals and the parafloccular volume [55], which shows similarities to cetaceans [56].

#### (ii) Nasal turbinates

Nasal turbinates have two functions, the anterior respiratory turbinates help with heat and water conservation, whereas the posterior part is involved in olfaction [57]. In carnivorans, the surface area of the turbinates can be correlated with habitat [58]. Aquatic taxa have large respiratory turbinates, but small olfactory ones, compared to terrestrial species. This helps to minimize heat loss in the water, where olfaction is less important. It has also been suggested that the nasal cavity plays a role in diet in carnivorans [59]. Larger canid and arctoid species have enlarged turbinals (the bones that enlarge the surface area) to locate prey items, compared to omnivorous species that are specialized on non-vertebrate food. Additionally, the paranasal system, located in the bones surrounding the nasal cavity, can fill spaces where bone is not necessary and allows modification in skull performance and minimizing mass [60].

#### (iii) Brain endocast

The brain endocasts can provide pivotal insights into the evolution of sensation and cognition in extant and extinct carnivorans. Much of the focus has been on relative brain size, which correlates with diet [61,62], home-range size [63], problem solving ability [64,65], type of parental care [66,67], hibernation [68] and extinction risk [69,70]. Evidently, there is a plethora of forces involved in the evolution of the brain, but there have been few attempts to integrate these various factors into a broader understanding of what influences brain evolution [71]. One recent study investigates brain size evolution across carnivorans and finds a trade-off between a relatively large brain and the costs of having a large brain for the individual when adapting to new environments [72]. An increasing number of studies have come to focus on the relative volumes of different regions of the brain [61,62,65,73–77] and have found correlations that were missed when looking at whole brain size. Enlargement of the frontal cortex correlates with sociality in hyenas, pantherines, canids and procyonids [61,65,73–76] in family-level analyses, but the trend is erased when sampling across the order. This highlights the importance of looking for such correlations at smaller taxonomic scales, such as family or subfamily level. For some time, the shape of the brain was widely neglected because of the lack of objective, quantitative methods to study and compare brain shape across species. Lyras *et al*. [78] examined brain folding and found that aquatic and semiaquatic carnivorans displayed more folded brains than terrestrial ones and that the degree of folding varied across carnivoran families. GMM has occasionally been used to analyse endocasts [79,80], which allows ecological inferences in extinct taxa [81], but remains an area for future development. GMM may be able to catch and pinpoint changes to the brain in subregions that cannot be isolated volumetrically and that are too minute to cause a significant volume change in the larger, volumetrically measurable region, such as the posterior cerebrum.

#### (iv) Eyes

Casares-Hidalgo *et al*. [82] investigated osteological correlates of visual strategy (i.e. nocturnal, diurnal and crepuscular) and other ecological variables such as habitat preference (i.e. open, close and mixed) and substrate use (i.e. terrestrial, arboreal and aquatic) from the orientation of the eye-socket. They concluded an absence of ecological correlates in the eye-socket of carnivorans and hypothesized that probably early Cenozoic representatives of stem ‘miacoids’ acquired a range of orbit angles that were useful for the entire range of ecologies exhibited by derived monophyletic groups of the crown-clade Carnivora. Therefore, the adaptation of different orbit orientations to specific ecological niches has simply been unnecessary [82]. Orbit orientation and size have recently been quantified in the sabretooth *Thylacosmilus atrox* (a South American sparassodont related to marsupials) to investigate its visual strategy [83]. Strikingly, authors found that *T. atrox* exhibited a peculiar divergent eye sockets with an unusual lack of visual convergence for a predator. Visual convergence entails stereoscopy, and this enhances the effectiveness of focus-and-follow behaviour [84]. Despite that, it has been recently proposed that *T. atrox* probably did not use its canines to dispatch its prey [85], but that its orbits were frontated and verticalized, and this compensated the limited convergence in orbit orientation [83].

### (c) Locomotion and hunting

Locomotor adaptation is an important way in which animals can exploit new resources, enter different environments and occupy new functional niches. Carnivores encompass a broad array of locomotor specializations including terrestrial and aquatic locomotion, and extensive climbing abilities [86]. Within terrestrial carnivores, there has been much interest in predatory behaviour as it can differentiate different types of predator–prey relationships. The postcranial skeleton has mainly been studied to understand extant carnivorans and to better understand extinct carnivorans [87]. Different aspects of their ecology are reflected in their limb morphology, where studies usually focus on locomotion, hunting and habitat (e.g. [87–89]) (figure 2).

#### (i) Limbs

Ecomorphological studies have always focused on hunting styles and type of prey capture [90,91]. Van Valkenburgh [91] found correlations between predatory behaviour, body size, metacarpal/phalanx ratio and femur/metatarsal ratio. Limb measurements can be associated with different hunting strategies [92–96] such as ambush, pursuit and pounce. Elbow morphology differentiates between pounce, ambush and pursuit predators [7,97,98], which in some cases can even overshadow a phylogenetic signal. Three-dimensional GMM on the forelimb [7,88,99,100] can distinguish between ambush, pounce and pursuit predators. Morphologies of extinct animals are occasionally found to fall outside of the range of modern carnivorans when it comes to hunting strategy [88,101], which implies modern species are not always fully comparable to extinct ones [88]. Habitat can also be inferred through ecomorphology [89,91,96,102,103]. It has been noted that the felid elbow can separate closed-habitat species from more open-habitat species [102]. This has also been studied to get an insight into palaeocommunities, where an open habitat environment was based on metacarpal length, with shorter lengths suggesting closed habitat, while longer ones would indicate an open habitat [91]. This can be correlated to the fact that closed habitat species exhibit more ambush behaviour, whereas open habitat species a more pursuit predation. Forelimb shape was both influenced by lifestyle and grasping ability in mustelids [100]. Species with a well-developed grasping ability have adaptations that tend to be exaggerated forms of arboreal adaptations. The pelvis shows a weak relationship between morphology and locomotor ecology, but a strong phylogenetic signal in carnivorans [104,105]. Similarly, the morphology of the hindlimb is mores strongly correlated with size and phylogeny with little influence of locomotion [105].

Ecomorphological studies of whether and how the distinct locomotor strategies of aquatic carnivores (i.e. pinnipeds) are reflected in their skeletons (e.g. coastal versus pelagic) are comparatively scarce (e.g. [106]). While phocid pinnipeds use pelvic oscillation and are usually classified as hindlimb-dominated swimmers [107], otariids use pectoral oscillation and are forelimb-dominated swimmers [108]. On the other hand, odobenids use an intermediate mode of locomotion, as their hindlimbs are the primary source of propulsion to generate thrust and their forelimbs are employed mainly for manoeuvring but also for propulsion [109]. Some studies have demonstrated an association between body proportions and different locomotory modes in extant pinnipeds [110], as well as in their vertebral morphology [111] and scaling [112], but GMM has rarely been applied.

#### (ii) Axial skeleton

The vertebral column plays a subtler role in organismal function and ecology, and its complexities present difficulties in analysis of ecomorphology. However, recent efforts have begun to elucidate the relationship between vertebrae morphology and ecology using GMM [113–118]. The anterior portion of the vertebral column in carnivores does not have significant ecomorphological signal [118]. Cervical vertebral morphology is highly constrained among carnivorans and has not been found to correlate with locomotor style or prey size [113,118]. Anterior thoracic vertebrae show weak signal for aquatic locomotion [111,118]. In otariid pinnipeds, anterior thoracic vertebrae are highly flexible, allowing for agility during swimming compared to more rigid spine in other carnivores [111]. This could suggest that the similar cervical and anterior thoracic morphology adequately functions for many different purposes, though further research on the ecomorphology of cervical and thoracic vertebrae is needed. The posterior portion of the vertebral column shows stronger ecomorphological signal in carnivores. Lumbar vertebrae have long been established as an important component of mammalian locomotion that facilitate the flexion and extension of the spine [119–121]. Among mammals, carnivores tend to be more dorsoflexible, incorporating more of this spinal flexion into locomotion, with the cheetah (*Acinonyx jubatus*) as an extreme example [114,120]. In all carnivorans, flat zygapophyses allow for this greater range of motion [122]. In more cursorial carnivores, long, anteriorly directed transverse processes and neural spines that increase the mechanical advantage of the epaxial muscles during locomotion [114]. Longer centra also increase the effective angle of bending in these taxa [121]. Alternatively, less cursorial taxa have less anteriorly inclined processes and shorter centra. For instance, *Ursus* has tall neural spines and long transverse processes, but both are perpendicular to the vertebrae [123]. Alongside shorter centra, these features increase the stability of the posterior lumbar region.

In carnivores, sacrum morphology is heavily influenced by phylogeny, but some ecomorphological signal demonstrates the functional significance of this portion of the spine [115]. The sacrum of carnivores correlates with locomotor style, body size and relative tail length. Carnivores with larger or more mobile tails showed an increase in muscle attachment area for the sacroccygeus and longissimus muscles. Tail length itself is associated with substrate, with arboreal mammals tending to maintain longer tails and tail loss generally occurring in terrestrial species [124]. Recently, the shape of the sacrum has been quantified using GMM in relation to the swimming strategies in extant pinnipeds [125]. The pinniped sacrum is characterized by a set of traits indicative of the low stresses it withstands because of the reduced time pinnipeds spent on land compared to terrestrial carnivorans. Moreover, they demonstrate clear morphological differences among the sacrum of phocids plus odobenids and otariids, which probably relates to their different modes of locomotion in both environments (i.e. land versus water) and not solely to their phylogenetic inheritance.

## 3. Forward perspective and synthesis

This review demonstrates that carnivoran ecomorphology is a flourishing research area which is providing remarkable insights into the relationship between ecology and morphology in mammals and vertebrates more broadly. When looking across the carnivoran skeleton, we find clues into many aspects of their ecology and behaviour (e.g. feeding, foraging, mating, locomotion, sensation), providing unique opportunities to potentially study ecological evolution through time in the fossil record (figure 2), and the broad application of phylogenetic comparative methods has provided new power to disentangle ecological and phylogenetic signals across species. However, several challenges remain.

### (a) Limitations of ecological classification in ecomorphology

While the scale and quality of morphological data has exploded with the recent innovation of high-throughput three-dimensional imaging (e.g. hand-held surface scanners), online morphology databases (e.g. phenome10k.org.; morphosource.org) and enhanced accessibility of zoological collections, our understanding of subtle and complex ecological differences among species has lagged behind, and inferences are often hampered by a lack of stable terminology [126]. For example, Samuels *et al*. [87] separate generalist ground-dwelling carnivorans and cursorial carnivorans into ‘terrestrial’ and ‘cursorial’ categories. All cursorial animals are already terrestrial, so cursoriality would be nested within terrestrial taxa. Another problem could be mixing different aspects of ecology, such as aquatic and fossorial [126]. Aquatic refers to habitat, while fossorial refers to locomotion. There are also no clear criteria for determining ecological types, with authors often only providing a reference to sources that describe ecology in a broader sense, especially in palaeontological research (e.g. [87,94,127]). Authors should be clearer on how exactly they decide an animal belongs to a certain ecological category based on the literature (e.g. [128]). Finally, it should be noted that ecological adaptations fall on a continuum [11]. It might be more beneficial to embrace this continuum, rather than sorting taxa into categories in which they do not always fit and that the concept of morphodynamic studies has also an influence on morphological evolution [10]. Increasing quantification of species-level ecological indicators, the compilation of broad data sources into online databases [129,130] and new technologies applied to animal behaviour (e.g. remote sensing) offer promising opportunities in this area [131]

### (b) Functional underpinnings and adaptive evolution

Ecomorphological inferences are primarily based on correlative relationships between morphology and behaviour with little quantitative analysis of the functional mechanisms which might underlie these adaptive responses. Though authors often speculate on the potential adaptive significance of various traits, there has been a historic separation between the ecomorphologists asking ‘what’ and biomechanists mostly asking ‘why’ [10,132]. However, recent analyses have used functional modelling of form–function relationships (e.g. finite-element analysis) to try to bridge this gap [133]. One excellent example is functional adaptive landscapes, which maps functional parameters onto morphological variation to understand functional differences between ecological groups [134]. Just as traditional adaptive landscapes examine the reproductive fitness of individuals based on their genotypes, so functional adaptive landscapes examine the functional optimization of species based on their morphology [134,135]. These landscapes have revealed the functional constraints and trade-offs which have shaped adaptive evolution in the turtle shell, or mammal vertebral column [135–137]. Application of these new techniques to carnivorans provides an opportunity to tease apart the functional adaptive signals represented by the ecological signals we find in the carnivoran skeleton. By combining GMM with biomechanical and phylogenetic comparative analysis, future work will provide a more holistic picture of carnivoran ecology and morphological evolution.

### (c) Mosaicism in ecomorphology: beyond single-element analysis

The majority of ecomorphological research focuses on single elements or simple ratios between elements, and there has been little consideration to how multiple elements of the skeleton may contribute together to produce holistic skeletal evolution. This is particularly challenging in GMM analyses, which are generally constrained to one-to-one comparisons of single elements [6]. While certain anatomical systems may be more or less involved in certain behaviours, it is clear that every organism must function as a whole and respond to their environment accordingly. This concept of morphological integration, or evolutionary covariation across skeletal elements, has been explored in depth across the bones of the skull, demonstrating that coordination between elements occurs across diverse vertebrate groups, including carnivores [138,139]. Semi-autonomous modules, with relative independence, may exist within the integrated organism, driven by developmental or functional links between elements, and may evolve and change through time [140]. Much work on this topic has focused on the skull, demonstrating evolutionarily conserved modules impact adaptive responses [138]. Such patterns have also been observed in the vertebral column of felids and carnivorans more broadly, demonstrating that modularity patterns can shape the adaptive response to ecology across systems [141–143]. However, the extent to which modularity and ecological adaptation interact broadly across skeletal elements is poorly understood.

Understanding the spread of ecological signal across the skeleton becomes even more important when considering ecomorphological inferences in the fossil record. The preservation of complete skeletons of extinct organisms is extremely rare. Instead, most fossils are characterized by a random selection of elements, that might range from a single isolated bone or tooth to a combination of separated or articulated elements with varying degrees of preservation. Given this fact, palaeoecological inferences are usually drawn from a subset of extinct species for which the relevant element of ecomorphological analysis is preserved. This restricts the sampling potential for the fossil record. However, exponentially increasing digitization efforts mean that it is within our grasp to gather data from multiple elements to better understand the interaction of ecomorphological signals across and between anatomical systems. Despite this, data analysis across multiple elements has been a barrier to progress. Most GMM approaches focus on single element comparisons using homologous landmarks. However, new approaches have recently been proposed for considering multiple elements [144]. For example, ‘Morphoblocks’ [145] examines variance–covariance matrices within and between multiple independent ‘blocks’ of landmarks to identify both common and unique morphological variation. Techniques such as these could be very powerful for testing ecomorphological signal across different combinations of elements, to facilitate extracting the maximum biological information from rare and precious fossil material.

### (d) Ecomorphological indicators in extinct species: towards solving the caveats of ecomorphology in studies of palaeobiology

Ecomorphological reconstruction of extinct species is usually performed from multivariate morphometrics on skeletal elements to test the association between the morphology of a given bone or tooth in the extant forms with their ecology or behaviour. Accordingly, morphological traits associated with known ecologies may be used as indicators of ecology and behaviour in extinct forms. However, the fact that ecomorphology is based on adaptive traits can also be a potential caveat for reconstructing the palaeoecology of extinct taxa using these kinds of methods. Ecomorphology can inform about the behaviour for which a trait is shaped by natural selection, but not on the actual behaviour engaged in. Therefore, while ecomorphology is useful to decipher adaptations through deep-time, it fails to decipher potential behavioural plasticity on shorter timescales. To solve this problem, it is necessary to combine ecomorphology with other approaches that capture aspects of behaviour during the animal's lifetime, such as dental wear analysis, which examines tooth damage caused during life [146,147], or isotopic biochemistry recorded in the fossilized bones and teeth [148,149]. These methods largely reflect lifetime habits rather than adaptive morphology [150]. Therefore, combining both types of analysis, it is possible to test how adaptive morphology matches with the actual lifetime habits of a given animal and to perform more accurate palaeoecological inferences for extinct taxa.

## Data Availability

This article has no additional data.
